# MRDtarget: A heuristic Gaussian approach for optimizing targeted capture regions to enhance Minimal Residual Disease detection

**DOI:** 10.1371/journal.pcbi.1013443

**Published:** 2025-09-17

**Authors:** Xuwen Wang, Yanfang Guan, Wei Gao, Xin Lai, Wuqiang Cao, Xiaoyan Zhu, Xiaoling Zeng, Yuqian Liu, Shenjie Wang, Ruoyu Liu, Xin Yi, Shuanying Yang, Jiayin Wang

**Affiliations:** 1 Department of Respiratory Medicine, The Second Affiliated Hospital of Xi’an Jiaotong University, Xi’an, Shaanxi, China; 2 School of Computer Science and Technology, Xi’an Jiaotong University, Xi’an, China; 3 Shaanxi Engineering Research Center of Medical and Health Big Data, Xi’an Jiaotong University, Xi’an, China; 4 Geneplus-Beijing, Beijing, China; Shenzhen University of Advanced Technology, CHINA

## Abstract

Molecular residual disease (MRD) detection, initially developed for hematologic malignancies, has become a critical biomarker for monitoring solid tumors. MRD detection primarily relies on circulating tumor DNA (ctDNA) analysis using next-generation sequencing, offering high sensitivity and broad genomic coverage. However, challenges remain in designing cost-effective panels that maximize mutation detection while maintaining biological relevance. Fixed panels often lack sufficient patient-specific mutation coverage, while WES-based personalized MRD assays, despite their high sensitivity, are costly and less accessible. We developed a tumor comprehensive genomic profiling (CGP)-informed personalized MRD assay to detect tumor-derived mutations, which allowed us to design patient-specific personalized panels and meanwhile, provide a cost-effective alternative to whole exome sequencing (WES). To address these limitations, we developed MRDtarget, a heuristic multivariate Gaussian model-based targeted capture region selection method. By expanding beyond traditional hotspot regions, MRDtarget optimizes variant tracking for MRD detection, significantly improving sensitivity. Using a Bayesian inference-based heuristic approach, MRDtarget integrates multi-feature informativeness rates to identify optimal genomic regions for capture. Experimental results demonstrate that MRDtarget enables the detection of more variants per patient. This study underscores the importance of rational panel design to improve MRD sensitivity and provides a novel approach to enhance precision diagnostics and treatment for solid tumor patients.

## Author summary

Minimal residual disease (MRD) detection plays a critical role in cancer prognosis and treatment monitoring, especially for solid tumors. However, existing sequencing panels often fail to provide sufficient coverage of tumor-specific mutations in every patient, limiting the clinical sensitivity of MRD detection. To address this gap, we developed MRDtarget, a computational tool that designs personalized targeted sequencing panels by optimizing the selection of genomic regions likely to contain informative mutations. Our method goes beyond conventional hotspot-based panels by incorporating multi-dimensional mutation features, including recurrence, clonality, and functional relevance, into a probabilistic model that prioritizes regions most informative for MRD detection. We evaluated MRDtarget using both clinical and public datasets and found that it consistently outperforms traditional approaches in mutation capture efficiency and patient coverage. Notably, it raises the proportion of patients with four or more trackable mutations—considered the minimum threshold for reliable MRD monitoring. MRDtarget also demonstrates robust performance across different cancer types and sequencing conditions. This approach enables a cost-effective and personalized solution for improving MRD detection, with broad implications for early relapse prediction and treatment guidance in precision oncology.

## 1. Introduction

Molecular residual disease (MRD), initially defined in hematologic malignancies, has been extended to solid tumors, where it serves as a valuable biomarker for recurrence risk and prognosis [1,2]. MRD detection primarily targets circulating tumor DNA (ctDNA) [3,4] or circulating tumor cells (CTCs) [5], helping clinicians identify high-risk patients early and guiding personalized treatment decisions [5,6]. Among detection methods, ctDNA mutation analysis using next-generation sequencing (NGS) has become the standard due to its high sensitivity, broad genomic coverage, and throughput. NGS-based ctDNA MRD detection is divided into fixed and personalized panel assays. Fixed panels, like CAPP-seq [7], are cost-effective and quickly deployable but have limited mutation coverage, particularly for rare cancers. WES-based personalized MRD assays, such as Signatera [8], use patient-specific tumor mutation profiles to achieve greater sensitivity and specificity with ultra-deep sequencing but are expensive and require tumor tissue sequencing. Tumor-informed strategies, which incorporate patient-specific mutation data, outperform tumor-agnostic approaches by reducing noise and improving detection. However, developing affordable, comprehensive genomic profiling (CGP) panels with sensitivity comparable to WES-MRD [9] remains a key challenge in advancing solid tumor MRD detection (detailed in the S1 Text).

The limit of detection (LOD) for ctDNA mutation-based MRD detection using NGS depends on factors such as cell-free DNA (cfDNA) input, sequencing depth, and the number of tracked mutations. Clinical MRD detection typically involves monitoring multiple specific mutations, significantly enhancing sensitivity and reliability. Increasing the number of tracked mutations under fixed cfDNA input and sequencing depth further reduces the LOD [10]. We developed a probability model based on binomial distribution theory, demonstrating that with 30 ng and 60 ng cfDNA input and four tracked mutations, the LOD reaches 0.02% (S1 Fig) and 0.01% (S2 Fig), which is consistent with the performance of the WES-customized MRD product Signatera that also achieves an LOD of 0.01%. However, traditional multi-gene panels often focus on a limited number of critical driver genes, limiting their sensitivity due to tumor heterogeneity [11]. Effective tissue-based panel designs must maximize detectable mutations in each patient’s tumor while maintaining the biological significance of mutation sites. This requires consistently detecting ctDNA at a frequency of ≥0.01% (with 60 ng cfDNA input) and achieving at least 95% patient coverage with four or more trackable mutation sites [8,12,13]. To meet these demands, novel algorithms are needed to enhance mutation detection capabilities, incorporate patient-specific mutation burdens, and maximize trackable mutations. These advancements will optimize MRD detection’s sensitivity and stability, addressing the challenges posed by tumor heterogeneity and detection complexity.

To enhance the sensitivity of MRD detection, we focus on panel design by optimizing targeted capture sequencing regions to increase the number of mutations tracked for MRD detection, thereby improving analytical performance. To achieve this, we developed a heuristic multivariate Gaussian model-based targeted capture region selection method, MRDtarget. The core functionality of MRDtarget lies in overcoming the limitations of traditional hotspot detection regions by screening exonic regions and expanding suitable genomic regions for detection. The process begins with manually selecting features of potential expansion regions based on expert knowledge. These features are then transformed into informativeness rates using a Poisson-Binomial model. Next, multi-feature informativeness rates are integrated to construct a Multivariate Gaussian Distribution model. Finally, an optimal set of expansion regions is identified using a heuristic approach based on Bayesian inference. Experimental results demonstrate that MRDtarget, compared to traditional multi-gene panel capture region selection methods, can more precisely and efficiently increase the number of mutations detected.

## 2. Materials and methods

**Fig 1:**
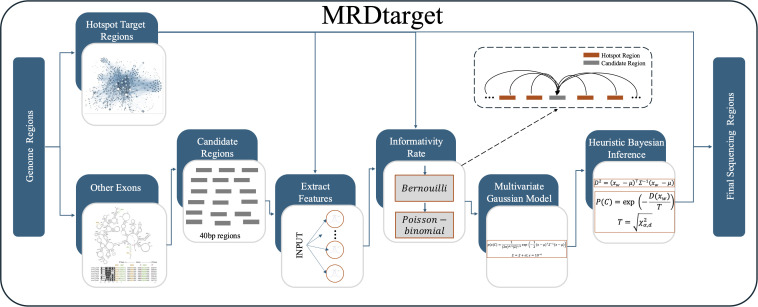
The workflow of MRDtarget.

We aim to enhance the detection rate of gene mutations by optimizing targeted capture sequencing regions, thereby improving the accuracy of MRD detection. This study focuses on two key aspects: first, optimizing mainstream hotspot detection region selection strategies; and second, proposing the screening of additional exonic regions beyond hotspot target regions. To achieve the expansion of detection regions beyond hotspots, this study introduces a heuristic algorithm based on Bayesian inference. The algorithm combines the probability density of a multivariate Gaussian distribution with Mahalanobis distance to identify and expand suitable genomic regions for detection. This ensures the scientific rigor and practicality of the expanded targeted capture sequencing regions (Fig 1).

### 2.1 Optimization of hotspot detection region selection

Currently, mainstream hotspot region selection strategies rely primarily on records from public databases, supplemented by manual review, to define targeted capture regions based on drug guidelines, clinical recommendations, and known hotspot regions. Building on this approach, this study further optimizes and expands the capture regions with a focus on improving the detection of exonic regions. The optimization process incorporates two key aspects: selecting exonic regions with high detection rates identified through Whole Exome Sequencing (WES) and targeting exonic regions with high detection rates within hotspot genes identified by WES.

1] Identifying Seed Regions: Seed genes are selected based on their relevance to prediction, prognosis, inheritance, diagnosis, or immunotherapy efficacy in the target cancer type. The selection prioritizes key cancer-related genes, including those approved by the NCCN, FDA, or NMPA, potential targets from literature reviews, and experimental targets currently in clinical trials listed on ClinicalTrials.gov (https://clinicaltrials.gov/).
2] Standardizing WES Gene Exon Intervals: A unique transcript is designated for each gene to ensure analytical consistency. Commonly referenced transcripts are chosen for documented genes, while the longest transcript in NCBI is used for others. Exon region coordinates are extracted from annotation files to form the WES gene exon set, which is refined by intersecting probe-covered regions from the training dataset with exon regions.
3] Feature Extraction Based on Exons: Patient data and corresponding WES variant sets are analyzed, sourced from clinical samples, published literature, and the TCGA database (https://portal.gdc.cancer.gov/). Metrics such as the number of patients with variants, total variants, and exon length are recorded. The Recurrence Index (**RI**) is calculated for each exon to prioritize target regions, representing the average number of variants per kilobase, as defined by Eq. (1).

$$RI=\text{1}\text{0}\text{0}\text{0}*\frac{n_{wp}}{n_{l}*n_{p}}$$ (1)

$n_{p}$ represents the number of patients in whom variants were detected in the exon region, i.e., the number of covered patients, **L** denotes the length of the exon detection region, and *n* is the total number of samples in the dataset. When counting the number of patients covered by the exon detection region, splice mutations (5’ or 3’ ends of exons) are included if the detection region contains the exon.

4) Selection of High Detection Rate Exon Regions: It involves sorting the Recurrence Index (**RI**) values in descending order to identify the most significant exon regions.

### 2.2 Screening of candidate regions for targeted sequencing based on a heuristic multivariate Gaussian model

In Section 2.1, we identified hotspot regions as the foundation for targeted capture region selection. Building on this, our study introduces a Bayesian inference-based heuristic algorithm to screen additional exon detection regions beyond the hotspots, expanding gene coverage and enhancing mutation detection. The algorithm (Table 1) integrates the probability density of a multivariate Gaussian distribution with Mahalanobis distance to identify high-priority exon regions for targeted sequencing. Core steps include feature extraction, multivariate Gaussian modeling, and heuristic screening via Bayesian inference.

**Table 1:** The algorithm of screening extended regions in targeted sequencing.

Algorithm: Method for Screening Extended Regions in Targeted Sequencing
Input: • Known target capture sequencing regions $X\in R^{n\times d}$: where *n* represents the number of data points and *d* represents the feature dimensions. • Candidate target capture sequencing regions to be evaluated $X_{t}\in R^{m\times d}$: where *m* is the number of points to be evaluated. • Significance level: α = 0.05. *• *Regularization term: $\in ={\text{10}}^{-\text{6}}$.
**Output:** Target capture sequencing expanded regions $R\in X_{t}$.
**Step 1: Compute Mean Vector and Covariance Matrix** • Compute the mean vector μ for the known target capture sequencing regions: $\begin{array}{c} \mu =\frac{\text{1}}{n}\sum_{i=\text{1}}^{n}\;x_{i} \end{array}$ • Compute the covariance matrix Σ for the known regions: $\begin{array}{c} \Sigma =\frac{\text{1}}{n-\text{1}}\sum_{i=\text{1}}^{n}\;\left(x_{i}-\mu \right){\left(x_{i}-\mu \right)}^{⊤} \end{array}$ • To ensure the invertibility of Σ, add a regularization term ∈ to its diagonal: Σ=Σ+∈I
**Step 2: Compute the Critical Value of the Chi-Squared Distribution** • Based on the feature dimensions *d*, calculate the critical value $\chi_{\text{threshold}}^{\text{2}}$: $\chi_{threshold}^{\text{2}}=\chi_{\alpha ,d}^{\text{2}}$ **Step 3: Compute Mahalanobis Distance** **• **For each candidate region $x_{w}\in X_{t}$, calculate the squared Mahalanobis distance: $D^{\text{2}}\left(x_{w}\right)={\left(x_{w}\;-\mu \right)}^{⊤}\Sigma^{-\text{1}}\left(x_{w}\;-\mu \right)$
**Step 4: Heuristic Bayesian Decision and Selection** • Initialize the target capture expanded regions set R=ϕ • For each candidate point $x_{w}\in X_{t}$: • if ${D\left(x_{w}\right)}^{\text{2}}\leq \chi_{\text{threshold}}^{\text{2}}$, add $x_{w}$ to R: $R=R\cup \{x_{w}\}$; • Otherwise, skip this point. **Step 5: Output** • Return the final set of expanded target capture sequencing regions R.

#### 2.2.1 Feature extraction.

MRDtarget begins by segmenting genomic regions to define the characteristics of exon detection regions. Specifically, for each exon, mutations within a 40 bp range are aggregated into a mutation cluster. The 40 bp window size was chosen based on typical probe design constraints in targeted sequencing panels, balancing region compactness with mutation clustering frequency. This window size is consistent with prior panel designs such as CAPP-Seq [7], where similar parameters are used to accommodate hybridization efficiency and regional mutation density. The region is then defined from the start position of the first mutation in the cluster to the end position of the last mutation. As a result, the length distribution of the regions includes mutation cluster regions of 40 bp or less. Based on this segmentation, the study extracts the following four key features from each 40 bp segment within the exon detection regions:

1) The WES Recurrence Index (**RI**): This metric evaluates mutation coverage in patients for each candidate detection region, representing the average number of variants per kilobase within the whole exome sequencing sample set. It is calculated using Eq. (1), with L = 40 as specified in Eq. (2).

$${RI}_{e}=\text{1000}*\frac{n_{wp}}{\text{40}*n_{p}}$$ (2)

${RI}_{e}$ represents the WES sample detection rate, $n_{wp}$ denotes the number of patients covered by the 40 bp region, and $n_{p}$ is the total number of samples.

2) Recurrence Improvement Index (RII): This metric evaluates the improvement in detecting additional variants for each candidate region, assessing its potential value in enhancing sample mutation coverage. When the total number of mutations covered by the existing regions is $N_{cover}$. Upon including the candidate region, the additional number of mutations covered for the sample is $N_{\text{40}bp-only}$. Assuming the designed target capture region ultimately aims to achieve N mutations covered per sample, the increase in the number of mutations detected in samples is defined as follows Eq. (3):

$$N_{add}=\left\{ \begin{array}{c} \text{0}\;,\;N_{cover}\geq N\\ N-N_{cover}\;,\;{N_{\text{40}bp-only}+N}_{cover}\geq N\\ N_{\text{40}bp-only}\;,\;{N_{\text{40}bp-only}+N}_{cover}<N \end{array}\right.\;$$ (3)

Recurrence Improvement Index (RII) is defined as Eq. (4):


$$RII=\frac{\sum_{i=\text{1}}^{n_{p}}N_{add}}{l_{e}}$$ (4)

**RII** represents the additional mutation detection contribution rate, $N_{add}$ denotes the number of additionally covered mutations, and $l_{e}$ represents the length of the additional probe region.

3) Clonal Recurrence Improvement Index (**Clonal RII**) measures the number of additional samples with clonal mutations detected by each candidate region, evaluating the potential value of the region in improving clonal mutation coverage. When the number of clonal mutations covered by the existing regions for a sample is $N_{cover-clonal}$, Upon including the candidate region, the additional number of clonal mutations covered for the sample is $N_{\text{40}bp-only-clonal}$. Assuming the designed target capture region ultimately aims to achieve **N** mutations covered per sample, additional Covered Clonal Mutation Count is defined as Eq. (5):

$$N_{add-clonal}=\left\{ \begin{array}{c} \text{0}\;,\;N_{cover-clonal}\geq N\\ N-N_{cover-clonal}\;,\;{N_{\text{40}bp-only-clonal}+N}_{cover-clonal}\geq N\\ N_{\text{40}bp-only-clonal\;}\;,\;{N_{\text{40}bp-only-clonal}+N}_{cover-clonal}<N \end{array}\right.\;$$ (5)

Clonal Recurrence Improvement Index (Clonal RII, RIIC) is defined as Eq. (6). Clonal mutations were defined based on cellular prevalence estimates using PyClone-VI. Specifically, variants in the cluster with maximum cellular prevalence were classified as clonal mutations. This threshold was selected to ensure robust representation of dominant tumor clones in downstream MRD analysis.


$${RII}_{C}=\frac{\sum_{\text{i}=\text{1}}^{n_{p}}N_{add-clonal}}{l_{e}}$$ (6)

4) The Functional Recurrence Improvement Index (Functional RII) was defined to evaluate the additional coverage contribution of candidate regions for functional (non-benign) mutations. When the number of non-benign (*nb*) mutations covered by the existing regions is $N_{cover-nb}$. Upon including the candidate region, the additional number of non-benign mutations covered for the sample is $N_{\text{40}bp-only-nb}$. Assuming the designed target capture region ultimately aims to achieve **N** mutations covered per sample, the increase in the number of functional (non-benign) mutations detected in samples is defined as follows Eq. (7):

$$N_{add-nb}=\left\{ \begin{array}{c} \text{0}\;,\;N_{cover-nb}\geq N\\ N-N_{cover-nb}\;,\;{N_{\text{4}\text{0}bp-only-nb}+N}_{cover-nb}\geq N\\ N_{\text{4}\text{0}bp-only-nb}\;,\;{N_{\text{4}\text{0}bp-only-nb}+N}_{cover-nb}<N \end{array}\right.\;$$ (7)

Functional Recurrence Improvement Index (Functional RII, RII_*f*_) is defined as Eq. (8):


$${RII}_{f}=\frac{\sum_{\text{i}=\text{1}}^{n_{p}}N_{add-nb}}{l_{e}}$$ (8)

#### 2.2.2 Feature informativity rate.

To facilitate the analysis, we introduce the concept of Feature Informativity Rate (**FIR**), which measures the performance of extracted features in candidate regions for targeted sequencing. Assume the extracted feature set is *f*, where $f_{i}$ represents the i-th feature, and the number of instances corresponding to this feature is *n* (*n*_*i*_ represents the number of instances for the i-th feature). The Informativeness is defined as $I_{ij}$, representing the informativeness of the j-th instance of the i-th feature. Specifically: $I_{ij}=\text{0}$: Non-informative instance, $I_{ij}=\text{1}$: Informative instance. $I_{ij}$ is assumed to be independent and follows a Bernoulli distribution (Eq. (9)), which varies across features. The number of informative instances surrounding $f_{i}$ is denoted as $n_{is}$. Notably, all hotspot detection regions mentioned in Section 2.1 are treated as informative instances. This definition of **FIR** helps quantify the contribution of different features in candidate regions to mutation detection, aiding in the systematic evaluation and optimization of targeted sequencing regions.


$$I_{ij}\sim Bernouilli\left(p_{ij}\right)$$ (9)

$$P\left(I_{ij}=\text{1}\right)=p_{ij},j=\text{1},\ldots ,n_{is}+\text{1}$$ (10)

$p_{ij}$ represents the informativity rate of the $j^{th}$ instance of the i-th feature. In the given feature set $f_{i}$, the total number of informative instances, $N_{i}$, is defined as Eq. (11):


$$N_{i}=\sum_{j=\text{1}}^{n_{is}+\text{1}}\;I_{ij},j=\text{1},\ldots ,n_{is}+\text{1}$$ (11)

The total number of informative instances, $N_{i}$, in feature $f_{i}$ represents the sum of all informative instances. Since the distribution of $I_{ij}$ varies, $N_{i}$ follows a Poisson-Binomial distribution. The cumulative distribution function (CDF) of $N_{i}$ is defined as Eq. (12):


$$F_{N_{i}}(l)=P(N_{i}\leq l),l=\text{0},\ldots ,n_{is}+\text{1}$$ (12)

We focus on the probability of having at least l informative instances in the feature set $f_{i}$. Specifically, the formula for calculating the feature informativity rate $f_{i}(l)$ is as follows Eq. (13):


$$f_{i}(l)=\text{1}-F_{N_{i}}(l-\text{1})$$ (13)

where, $f_{i}(l)$ represents the probability that the feature set $f_{i}$ contains at least l informative instances; $F_{N_{i}}(l-\text{1})$) is the cumulative distribution function (CDF) of $N_{i}$, the total number of informative instances in $f_{i}$. The default value for l is set to $n_{is}$+1, where $n_{is}$denotes the number of informative instances in the surrounding region.

Based on the above definition, a feature informativity rate vector $f_{i}$ can be computed for each candidate region for targeted sequencing Eq. (16):


$$f_{i}=\{f_{i\text{1}},f_{i\text{2}},...,f_{im}\}$$ (14)

where: *m* represents the total number of features in the candidate region for targeted sequencing. It should be noted that the Poisson-Binomial model assumes conditional independence among feature instances. While features such as RI and RIIC may exhibit mild correlations, we adopt this assumption for tractability and interpretability. Future extensions may consider dependency-aware models.

#### 2.2.3 Multivariate Gaussian modeling.

Based on the analysis in Section 2.2.2, feature informativity rates for each candidate region in targeted sequencing were obtained. According to probabilistic statistical theory, the feature informativity rates for each dimension approximately follow a Gaussian distribution [14]. Therefore, the overall feature informativity rates can be modeled using a Multivariate Gaussian Distribution, with the probability density function defined as Eq. (15):


$$p(x|C)=\frac{\text{1}}{(\text{2}\pi {)}^{d/\text{2}}|\Sigma {|}^{\text{1}/\text{2}}}exp\left(-\frac{\text{1}}{\text{2}}(x-\mu {)}^{⊤}\Sigma^{-\text{1}}(x-\mu )\right)$$ (15)

where:

- *d* refers to the number of features used to represent each candidate region in the informativity vector *x*
- $x\in R^{d}$: A d-dimensional feature vector representing a candidate region to be evaluated.
- $\mu \in R^{d}$: The mean vector, representing the center of the known capture sequencing region distribution C.
- $\Sigma \in R^{d\times d}$: The covariance matrix, representing the relationships between features.
- |Σ|: The determinant of the covariance matrix.
- $\Sigma^{-\text{1}}$: The inverse of the covariance matrix.

To ensure the numerical stability and invertibility of the covariance matrix Σ, a small regularization term ∈ (default value $\in ={\text{10}}^{-\text{6}}$) is added to its diagonal elements. The definition is Eq. (16):


$$\Sigma =\Sigma +\in I,\in ={\text{10}}^{-\text{6}}$$ (16)

***I*** is the identity matrix. Regularization techniques are widely used to address potential singularity issues in covariance matrices and are broadly applied in multivariate Gaussian modeling [15].

The multivariate Gaussian distribution is uniquely determined by the mean vector μ and the covariance matrix Σ. μ is to describe the central location of the distribution, reflecting the average feature informativity rates of known capture sequencing regions. Σ is to capture the correlations between features, helping to comprehensively characterize the statistical properties of candidate regions. For a candidate region $x_{w}$, its conformity to the distribution characteristics of known capture sequencing regions can be assessed by computing its probability density $p(x_{w}\;)$ under the distribution **C**. If $p(x_{w}\;)$ is high, the candidate region is close to the central characteristics of the known distribution and can be considered a potential expansion region for targeted sequencing. If $p(x_{w}\;)$ is low, the candidate region significantly deviates from the known distribution and can be regarded as an outlier, unsuitable for expansion.

#### 2.2.4 Heuristic screening based on bayesian inference.

To estimate the prior probability of a candidate exon region $x_{w}$, we adopt a Bayesian-inspired heuristic approach that leverages the Mahalanobis distance from $x_{w}$ to the centroid of known capture regions. This distance is transformed into a probability score using an exponential decay function, where smaller distances yield higher probabilities, indicating closer similarity to the known distribution. Although this transformation deviates from classical Bayesian priors, it effectively captures the centrality of data points within a multivariate Gaussian framework. Similar strategies have been applied in functional data classification (e.g., Galeano et al. [16]), and anomaly detection using local Mahalanobis distances (e.g., Yang et al. [17]) and weighted Mahalanobis models (e.g., Wen et al. [18]). The squared Mahalanobis distance $D^{\text{2}}$ is calculated as Eq. (17):


$$D^{\text{2}}={\left(x_{w}\;-\mu \right)}^{⊤}\Sigma^{-\text{1}}\left(x_{w}\;-\mu \right)$$ (17)

Here, *µ* is the mean vector of known target regions, and *Σ* is the covariance matrix. A small $D^{\text{2}}$ indicates that $x_{w}$ is close to the distribution center, suggesting suitability as an expanded capture region. Conversely, a large $D^{\text{2}}$ implies outlier status.

The prior probability *P*(*C*) for a candidate region is computed via Eq. (18):


$$P(C)=exp\left(-\frac{D(x_{w})}{T}\right)$$ (18)

Where T is a critical value derived from the Chi-Squared distribution, used for normalizing the distance and ensuring the prior probability lies within the range [0, 1]. And T is calculated based on the significance level α and degrees of freedom *d* (number of features) using Eq. (19). The use of exponential decay to transform Mahalanobis distance into a prior probability is inspired by its effectiveness in probabilistic outlier detection. This transformation allows soft penalization of outlier regions while preserving a continuous scoring spectrum. Similar approaches have been used in functional data classification (e.g., Galeano et al.) [16], where distance-based scores are mapped into probability estimates to reflect centrality in the feature space.


$$T=\sqrt{\chi_{\alpha ,d}^{\text{2}}}$$ (19)

Under the assumption of multivariate normality, the squared Mahalanobis distance ${D(x_{w})}^{\text{2}}$ follows a Chi-Squared distribution with degrees of freedom *d*. Therefore, outliers can be determined using the critical value of the Chi-Squared distribution. Given a significance level α (set to 0.05 by default) and the number of features *d*, the critical value is calculated using Eq. (20):


$$\chi_{threshold}^{\text{2}}={ChiSquared}^{-\text{1}}(\text{1}-\alpha ,d)$$ (20)

Here, ${\text{ChiSquared}}^{-\text{1}}$ is the inverse cumulative distribution function of the Chi-Squared distribution. When ${D(x_{w})}^{\text{2}}\leq \chi_{\text{threshold}}^{\text{2}}$, $x_{w}$ belongs to the known distribution and can be considered as a potential extension region for targeted sequencing. Conversely, when ${D(x_{w})}^{\text{2}}\leq \chi_{\text{threshold}}^{\text{2}}$, $x_{w}$ is considered an outlier and unsuitable as an extension region.

## 3. Results

### 3.1. Sample preparation

To validate the effectiveness of MRDtarget, its performance was analyzed and evaluated using both public database samples and clinical samples. The validation compared WES results with targeted capture sequencing results obtained using a conventional pre-optimized panel, hereafter referred to as Tdesigner, and the optimized panel generated by our proposed algorithm, MRDtarget. Tdesigner was constructed based on standard hotspot region selection strategies, relying on public database records and clinical guidelines without incorporating recurrence-based metrics or statistical screening. In contrast, MRDtarget integrates WES-derived recurrence features and a Bayesian heuristic model to expand the capture region beyond known hotspots. Key performance metrics, including variant density and clonal population clustering, were used to comprehensively assess the performance improvements achieved by MRDtarget over Tdesigner.

#### 3.1.1. TCGA sample cohort.

This study utilized 1,024 lung cancer samples from The Cancer Genome Atlas (TCGA) database, sequenced using Whole Exome Sequencing. The cohort included 544 lung adenocarcinoma cases (TCGA Project ID: TCGA-LUAD) and 480 lung squamous cell carcinoma cases (TCGA Project ID: TCGA-LUSC), representing the pathological subtypes of lung cancer. By comparing variant detection results from WES, pre-optimized, and post-optimized targeted capture panels, the study aimed to comprehensively assess the sensitivity and practical utility of the optimized panel design.

#### 3.1.2. Clinical sample cohort.

We also included 150 clinical solid tumor tissue samples, comprising 123 lung cancer cases, 10 breast cancer cases, 8 digestive system tumor cases, and 9 cases of other solid tumors. Each sample was analyzed using both pre-optimized and post-optimized targeted capture panels, with sequencing depth exceeding 500x. Variant counts were compared between the two panels for the same samples. PyClone-VI [19] was subsequently employed to conduct clustering analysis on the detected variants, enabling the classification of clonal and sub-clonal variants.

#### 3.1.3. Preprocessing.

Raw sequencing data were preprocessed by trimming terminal adaptors and removing low-quality sequences, defined as those with more than 50% N bases or more than 50% of bases having a quality score (Q) <5. The clean reads were aligned to the reference human genome (GRCh37.p13, GCF_000001405.25) using BWA-MEM2 (version 2.2.1a) [20]. Patient-specific somatic variants were identified by analyzing sequencing data from primary tumors and matched peripheral blood lymphocyte (PBL) samples. Tumor somatic single-nucleotide variants (SNVs) and small insertions and deletions (InDels) were called using RealDcaller2 (version 2.0.9) and TNscope (v3.8.0; Sentieon Inc.), as described previously [21,22]. Structural variants (SVs) were profiled using NCsv2 (version 1.2.0), an in-house tool developed at Geneplus-Beijing [21,22].

### 3.2. Performance on TCGA samples

#### 3.2.1 Variant density.

Existing studies have demonstrated that increasing the number of variant monitoring sites can lower the limit of detection (LoD) and enhance the sensitivity of MRD detection [23]. In this study, variants from the TCGA dataset were filtered by excluding those with Variant_Classification values of RNA, 3’Flank, 5’Flank, or intergenic region (**IGR**), as well as mutations located outside the ± 50 bp range of intron regions. To evaluate the effectiveness of the proposed MRDtarget tool, we compared the optimized method with WES across TCGA samples. Variant density for both MRDtarget and WES was calculated, with the variant density (*d*), representing the average number of variants per kilobase, as defined by Eq. (21):


$$d=\text{1}\text{0}\text{0}\text{0}*\frac{n_{m}}{l_{r}}$$ (21)

**Fig 2:**
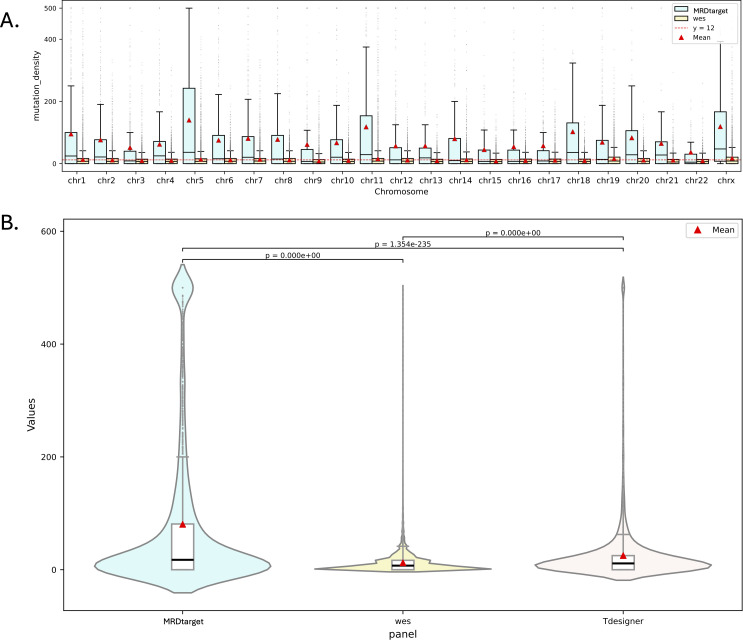
Comparison of Variant Density Distributions Across Methods. Variant density distributions across all chromosomes (excluding the Y chromosome) are shown for MRDtarget, Tdesigner, and WES. (A) Variant densities for each targeted capture region were calculated and visualized, highlighting the improved performance of MRDtarget compared to WES. (B) A comparison of the variant density distributions among MRDtarget, Tdesigner, and WES demonstrates their distinct gene detection strategies. MRDtarget consistently achieves higher variant densities across autosomes and the X chromosome, emphasizing its optimized capture capability.

Here, d represents the variant density, $n_{m}$ is the number of variants (sourced from a publicly available variant dataset) detected within the targeted capture region, and $l_{r}$ is the length of the targeted capture region. Variant density quantifies the effectiveness of targeted sequencing methods in detecting variants, with higher values indicating enhanced detection capabilities. We calculated and visualized the variant density across all chromosomes (excluding the Y chromosome) for each targeted capture region (Fig 2A). MRDtarget, representing the optimized method, showed a significant improvement over WES, achieving a 5.67-fold and 1.57-fold increase in mean and median variant density, respectively. This demonstrates the superior variant capture capability of MRDtarget, consistently outperforming WES across all autosomes and the X chromosome.

To evaluate the performance of MRDtarget against traditional panel design strategies, we compared the variant densities of MRDtarget, Tdesigner, and WES (Fig 2B). The significantly distinct variant density distributions (p < 0.01) among the three methods highlight their differing gene detection and capture strategies. MRDtarget outperformed Tdesigner, achieving a mean variant density of 80 versus 25 and a median of 18 versus 10, representing 2.20-fold and 0.8-fold improvements, respectively. Both MRDtarget and Tdesigner showed higher variant densities than WES, with MRDtarget achieving 5.67-fold and 1.57-fold increases in mean and median densities, while Tdesigner achieved 1.083-fold and 0.429-fold improvements. These results emphasize the superior performance of MRDtarget in enhancing variant detection by focusing on high-variant-density regions, providing precise genomic insights for applications such as precision oncology and MRD detection. Further optimization of Tdesigner, incorporating data-driven strategies, could enhance its ability to prioritize key genomic regions.

### 3.3. Performance on clinical cancer samples

We analyzed 150 clinical solid tumor tissue samples, including 123 lung cancer cases, 10 breast cancer cases, 8 digestive system tumor cases, and 9 cases of other solid tumors, using both pre-optimized (Tdesigner) and post-optimized (MRDtarget) targeted capture panels with sequencing depths exceeding 500x. Previous studies [12,13] have emphasized the need for NGS-based ctDNA multi-gene panels to comprehensively cover class I/II gene variants and reliably detect ctDNA at abundances ≥0.01% with 60 ng of cfDNA. Such panel designs must increase the number of detectable tumor tissue variants per patient while ensuring that 95% of patients have four or more monitorable variant sites, thereby enhancing MRD detection sensitivity. Variant counts between the two panels were compared, and clonal population clustering was conducted using PyClone-VI to annotate clonal and sub-clonal variants.

#### 3.3.1 Variant counts.

**Fig 3:**
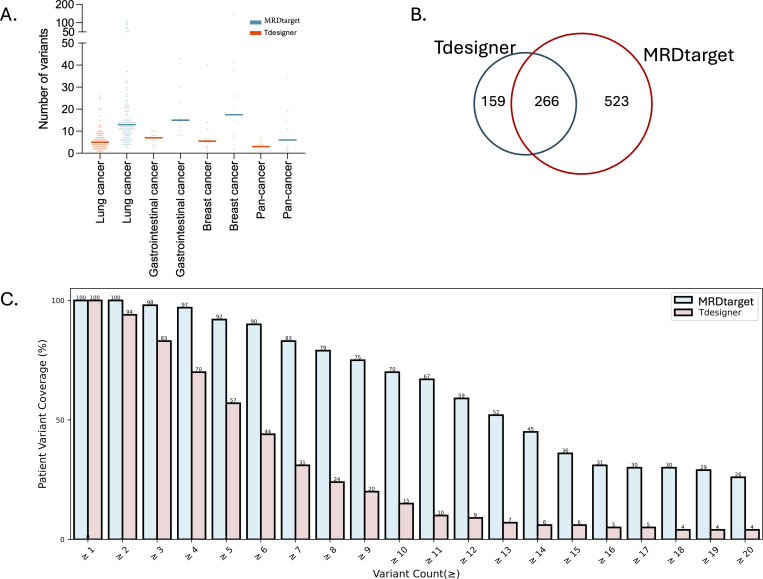
The performance on variant detection. (A) Median variant counts detected by MRDtarget and Tdesigner across 123 lung cancer cases, 10 breast cancer cases, 8 digestive system tumor cases, and 9 other solid tumors. (B) Comparison of clonal variants detected by MRDtarget and Tdesigner. (C) Patient coverage analysis for MRDtarget and Tdesigner.

We first evaluated the performance of MRDtarget and Tdesigner in detecting variants across different cancer types. As shown in Fig 3A, MRDtarget demonstrated superior variant detection capabilities compared to Tdesigner across all tumor types. The median variant counts detected by MRDtarget and Tdesigner in 123 lung cancer cases, 10 breast cancer cases, 8 digestive system tumor cases, and 9 other solid tumor cases were (13, 15, 17.5, 6) and (5, 7, 5.5, 3), respectively. These results highlight MRDtarget’s enhanced ability to identify variants across diverse tumor types, emphasizing its robustness and reliability for broader clinical applications. Next, we compared the performance of MRDtarget and Tdesigner in detecting clonal variants. As shown in Fig 3B, Tdesigner identified 425 clonal variants, while MRDtarget detected 789. In 64.7% of the samples (97 out of 150), MRDtarget identified more clonal variants than Tdesigner, with a median increase of three variants per sample. These findings demonstrate that the optimized MRDtarget panel significantly improves the detection of clonal variants. The ability to detect more clonal variants highlights MRDtarget’s improved sensitivity and efficiency, which are critical for MRD monitoring. Clonal variants are closely associated with disease progression and treatment outcomes, making MRDtarget a more effective tool for reliable MRD assessments. Finally, we analyzed the patient coverage achieved by MRDtarget and Tdesigner under a fixed number of mutations. As shown in Fig 3C, MRDtarget achieved 97% patient coverage for those with four or more trackable mutation sites, exceeding the 95% threshold required to maximize population coverage and MRD detection sensitivity [8,12,13]. In comparison, Tdesigner only covered 70% of patients meeting this criterion. From a clinical perspective, achieving four or more trackable mutations per patient is often considered a critical threshold for reliable MRD monitoring. Our optimized MRDtarget achieves this in 97% of patients, exceeding the 95% benchmark suggested in prior studies [8,12,13].

#### 3.3.2 Clonal variant counts.

**Fig 4:**
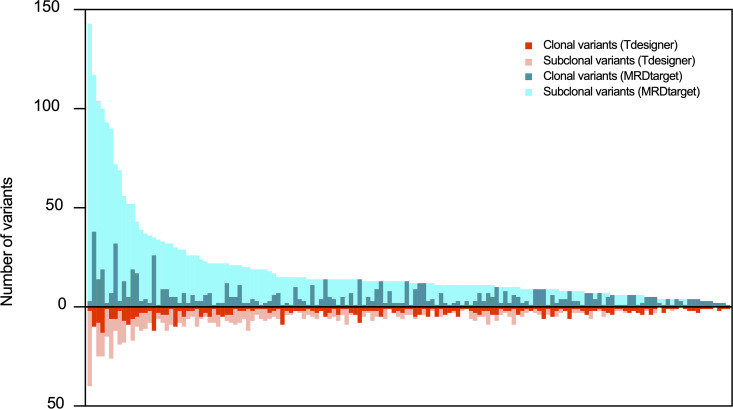
The performance on clonal variants. Each bar represents a single patient, with the top portion indicating the number of major clonal variants and the bottom portion representing subclonal variants detected using MRDtarget and Tdesigner.

Study [23] has shown that clonal variants hold greater prognostic value than subclonal variants in MRD monitoring. In our experiment with 150 clinical cancer samples, as illustrated in Fig 4, each bar represents a single patient. The top portion of each bar shows the number of clonal variants detected, while the bottom portion indicates the subclonal variants, using MRDtarget and Tdesigner for the same patients. A comparison demonstrates that MRDtarget consistently detects a higher total number of variants for most patients, with notable increases in both clonal and subclonal variants. The improved detection of clonal variants aligns with MRDtarget’s design goal of prioritizing clinically significant regions, while the enhanced detection of subclonal variants reflects its broader sensitivity to low-abundance variants. These advancements are particularly significant for MRD monitoring, where comprehensive variant detection is essential for accurately assessing tumor burden and disease progression. By identifying more variants across both categories, MRDtarget generates a more detailed and reliable genomic profile, enhancing patient stratification and supporting the development of personalized therapeutic strategies.

## 4. Discussion and conclusion

MRDtarget is an advanced tool for optimizing targeted capture sequencing regions in MRD detection. By integrating a heuristic multivariate Gaussian model, MRDtarget overcomes key limitations of traditional panel design strategies, such as limited patient-specific mutation coverage and reduced sensitivity. By expanding beyond hotspot regions and incorporating high-density exonic regions, MRDtarget enhances its utility across diverse cancer types. Compared to Tdesigner and WES, MRDtarget demonstrates superior performance, achieving significantly higher variant densities and providing better coverage of clonal and subclonal variants. Notably, it consistently outperforms Tdesigner in patient coverage, with 97% of patients having four or more trackable mutation sites, exceeding the 95% threshold for optimal MRD detection. Its ability to detect more clonal variants, which are critical for disease progression and treatment decisions, enhances the precision of MRD assessments and supports personalized therapeutic strategies. MRDtarget’s tailored capture strategies address tumor heterogeneity by prioritizing regions likely to harbor key variants, enabling a more detailed genomic profile. This is essential for applications in biomarker discovery, precision oncology, and genetic research. The use of Bayesian inference and multivariate Gaussian modeling provides a reliable statistical foundation, ensuring accuracy and reproducibility. All features are currently standardized and treated equally, with no additional weighting applied. However, we would like to emphasize that in the heuristic screening stage based on Bayesian inference, the final selection of candidate regions is guided by their similarity to known hotspot regions. These hotspot regions themselves are determined using conventional panel design strategies, which often prioritize regions with high RI values. As a result, although we do not explicitly assign weights to individual features, the upstream selection of hotspot regions may implicitly introduce a bias that favors certain features—particularly RI—during the final region selection process. However, the tool’s reliance on existing features for region selection highlights an area for improvement. Expanding the feature set could enhance its robustness and adaptability across diverse cancer types and genomic contexts. Future efforts will focus on expanding the feature set to further improve precision and applicability. In addition, we plan to conduct a sensitivity analysis to quantitatively assess the relationship between probe length, sequencing cost, and detection sensitivity. This will enable a more balanced design of capture panels that optimize both efficiency and clinical feasibility. Although the current analysis was based on the hs37d5 (GRCh37.p13) reference genome, chosen for its compatibility and widespread use in clinical pipelines, we acknowledge its limitations in handling sex-specific variation. Notably, Y chromosome variants were not explicitly excluded during downstream analysis. This design choice aimed to ensure broad applicability across mixed-sex populations. As a result, features from X- and Y-linked regions were calculated uniformly, without sex-specific weighting. While this may reduce precision in certain scenarios, it simplifies implementation across heterogeneous cohorts. Following best practices in sex-aware genomic analysis (Olney et al., 2020) [24], future versions of MRDtarget will adopt sex-specific reference genomes based on GRCh38.p14 and incorporate population-stratified modeling strategies to improve the accuracy, robustness, and equity of variant detection.

In conclusion, MRDtarget represents a significant advancement in MRD detection, offering an efficient and sensitive framework for targeted sequencing panel optimization. MRDtarget can push more patients above the four-variant threshold further underscores its potential to improve real-world MRD detection coverage. And its strong performance in variant detection and patient coverage underscores its potential for advancing precision oncology and improving clinical outcomes. With further refinement and validation, MRDtarget could play a central role in developing cost-effective, high-performance genomic profiling panels for cancer diagnosis and treatment.

## 5. Key points

- Novel Heuristic Model: MRDtarget integrates a multivariate Gaussian model with Bayesian inference to optimize targeted capture region selection, achieving higher variant densities and ensuring 97% patient coverage with four or more trackable mutations.
- Expansion of Exonic Regions: Focuses on high-density exonic regions to enhance variant detection beyond traditional hotspot strategies.
- Iterative Refinement of Target Regions: Employs a systematic process of feature extraction, statistical modeling, and heuristic optimization to iteratively refine and expand capture regions for enhanced variant detection.

## Supporting information

S1 Text Supplementary text and Supplementary Figures. (DOCX)

S1 Fig 30ng cfDNA. (TIF)

S2 Fig 60ng cfDNA. (TIF)
