# Supplementary material for: MRDtarget: A heuristic Gaussian approach for optimizing targeted capture regions to enhance Minimal Residual Disease detection
Source: PLoS Comput Biol. 2025 Sep 17;21(9):e1013443. doi: 10.1371/journal.pcbi.1013443 (PMC12456828; doi:10.1371/journal.pcbi.1013443)
Supplement: S1 Text — (DOCX) [file pcbi.1013443.s001.docx]

## **Supplementary Materials**

**1. Strategies and Advances in ctDNA-Based MRD Detection for Solid Tumors**

The development of ctDNA-based MRD detection strategies for solid tumors using NGS involves two critical considerations: whether to design personalized probes or primers for each patient and whether to incorporate tumor tissue mutation information into the analysis. Based on the approach to probe or primer design, MRD detection can be categorized into personalized assays and fixed panel assays. Fixed panel assays are constructed based on large genomic datasets of cancer populations and are designed to detect driver genes, frequently mutated genes, and genes related to resistance and tumor evolution. These assays, such as CAPP-seq (7), offer ready-to-use panels for all patients, enabling convenient and rapid clinical application as well as monitoring of acquired mutations driven by tumor evolution. However, fixed panels have limited mutation coverage for individual patients, with a median of four SNVs per patient, and typically operate at a lower sequencing depth compared to personalized approaches (10,000X vs. 100,000X). This limitation can affect the sensitivity of MRD detection (25). Additionally, fixed panel assays are less suitable for rare cancer types due to suboptimal patient coverage. In contrast, personalized MRD assays, such as Signatera (8) and the Invitae personalized cancer monitoring test using patient-specific Anchored Multiplex PCR (AMP) technology (26, 27), leverage ultra-deep sequencing (100,000X) to specifically detect sufficient numbers of tumor-derived mutations, thereby improving the sensitivity for detecting true tumor mutations. Personalized assays require initial tumor tissue sequencing to design patient-specific probes or primers tailored to the tumor’s mutation profile. WES-based personalized MRD assays are renowned for their exceptional performance but are associated with high costs. A recent head-to-head comparison of MRD detection in bladder cancer patients demonstrated that a tumor comprehensive genomic profiling (CGP)-informed personalized ctDNA assay achieved comparable results to WES-based assays while significantly reducing costs (9). From the perspective of tumor information inclusion, MRD detection can be divided into tumor-informed and tumor-agnostic/naïve strategies. Tumor-agnostic/naïve approaches do not rely on patient-specific tumor mutation information, which can compromise their specificity and sensitivity (28). In contrast, tumor-informed strategies incorporate the patient’s tumor mutation profile into bioinformatic analyses, effectively reducing background noise and false-positive results, thereby improving performance. Retrospective studies have demonstrated that tumor-informed strategies are significantly more effective than tumor-agnostic/naïve approaches in MRD detection (12, 25). Many MRD detection products that have received breakthrough device designations from the U.S. FDA are based on tumor-informed, personalized strategies. Given the current technological and clinical evidence, tumor-informed analyses and personalized assay products have become the mainstream approach for solid tumor MRD detection.

**2. Theoretical Derivation of ctDNA LOD**

The limit of detection (LOD) of circulating tumor DNA (ctDNA) is a critical metric for evaluating the performance of molecular residual disease (MRD) detection. Based on the binomial distribution theory, this study derives a probability model for ctDNA detection and analyzes the performance of ultra-sensitive detection under specific conditions, considering the input cfDNA amount, variant allele frequency (VAF), and the number of tumor-specific somatic variants.

The probability *p* of detecting at least one ctDNA molecule can be expressed as:

$$p=1-e^{-n\cdot f\cdot m}$$

where:

- *n* represents the number of effective molecules, influenced by the cfDNA input amount and nucleic acid template conversion efficiency.
- *f* indicates the ctDNA level (i.e., VAF, defined as the ctDNA % at the LOD).
- *m* denotes the number of tumor-specific somatic mutations (specifically the number of unique variant sites used for detection in this study).

This formula assumes that ctDNA molecules in the input cfDNA follow a binomial distribution and are approximated by a Poisson distribution. As the cfDNA input nn and the number of mutations mm increase, the detection probability pp improves, enabling lower detection limits *f*.

**Example Analysis**

With a 30 ng cfDNA input (equivalent to approximately 9,000 haploid genome molecules) and monitoring four mutations, an LOD of 0.02% can be achieved, aligning with the performance metrics proposed in the "Expert Consensus on Molecular Residual Disease of Non-Small Cell Lung Cancer." Furthermore, with a 60 ng cfDNA input (equivalent to approximately 18,000 haploid genome molecules) and monitoring the same four mutations, the LOD can be improved to 0.01%, matching the sensitivity level of the international benchmark product Signatera WES-MRD. These results demonstrate that increasing cfDNA input and optimizing the number of variant sites can significantly enhance ctDNA detection performance.

**Key Parameters in Model Derivation**

Effective Molecule Count (*n*): Determined by the cfDNA input amount and template conversion efficiency. In this study, 1 haploid genome molecule is assumed to be present per 3.3 pg of cfDNA. Thus, 30 ng and 60 ng of cfDNA correspond to approximately 9,000 and 18,000 molecules, respectively. ctDNA Level (*f*): Defined as the minimum detectable ctDNA percentage. Theoretically, f can be reduced by adjusting the input amount and the number of mutations. Number of Somatic Mutations (*m*): A positive correlation exists between the number of monitored mutations and detection sensitivity. Although Signatera products typically monitor 16 mutations, this study demonstrates that even with only four mutations, comparable performance can be achieved by increasing the cfDNA input. This study validates that lowering the ctDNA LOD can be effectively achieved by increasing cfDNA input and optimizing the number of monitored mutations. For instance, under the conditions of 30 ng and 60 ng cfDNA input with four monitored mutations, LODs of 0.02% and 0.01%, respectively, can be achieved (as illustrated **S1** **Fig** and **S2 Fig**).
