# Supplementary figures and images for: MRDtarget: A heuristic Gaussian approach for optimizing targeted capture regions to enhance Minimal Residual Disease detection
Source: PLoS Comput Biol. 2025 Sep 17;21(9):e1013443. doi: 10.1371/journal.pcbi.1013443 (PMC12456828; doi:10.1371/journal.pcbi.1013443)

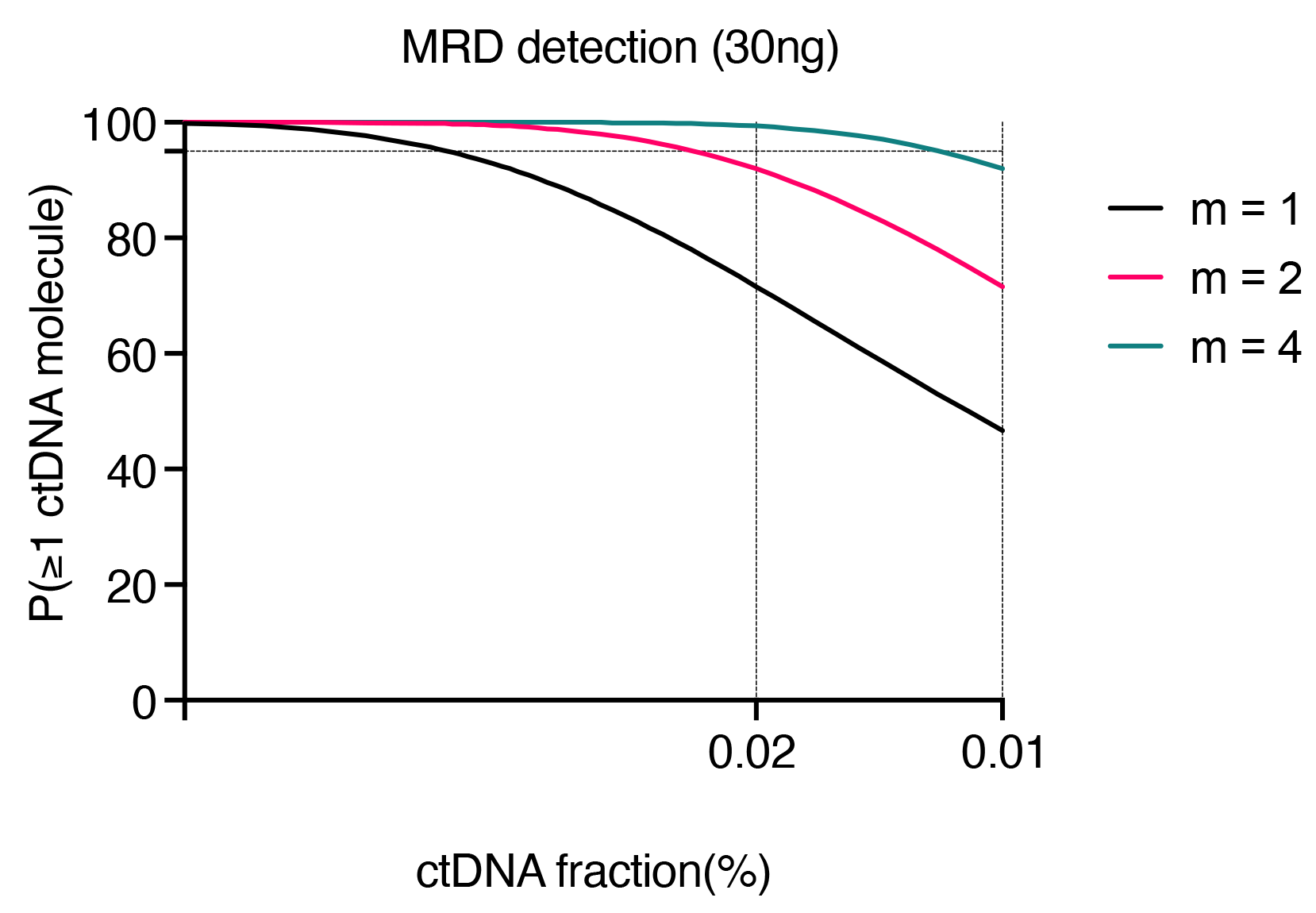

Supplement: S1 Fig — (TIF) [file pcbi.1013443.s002.tif]

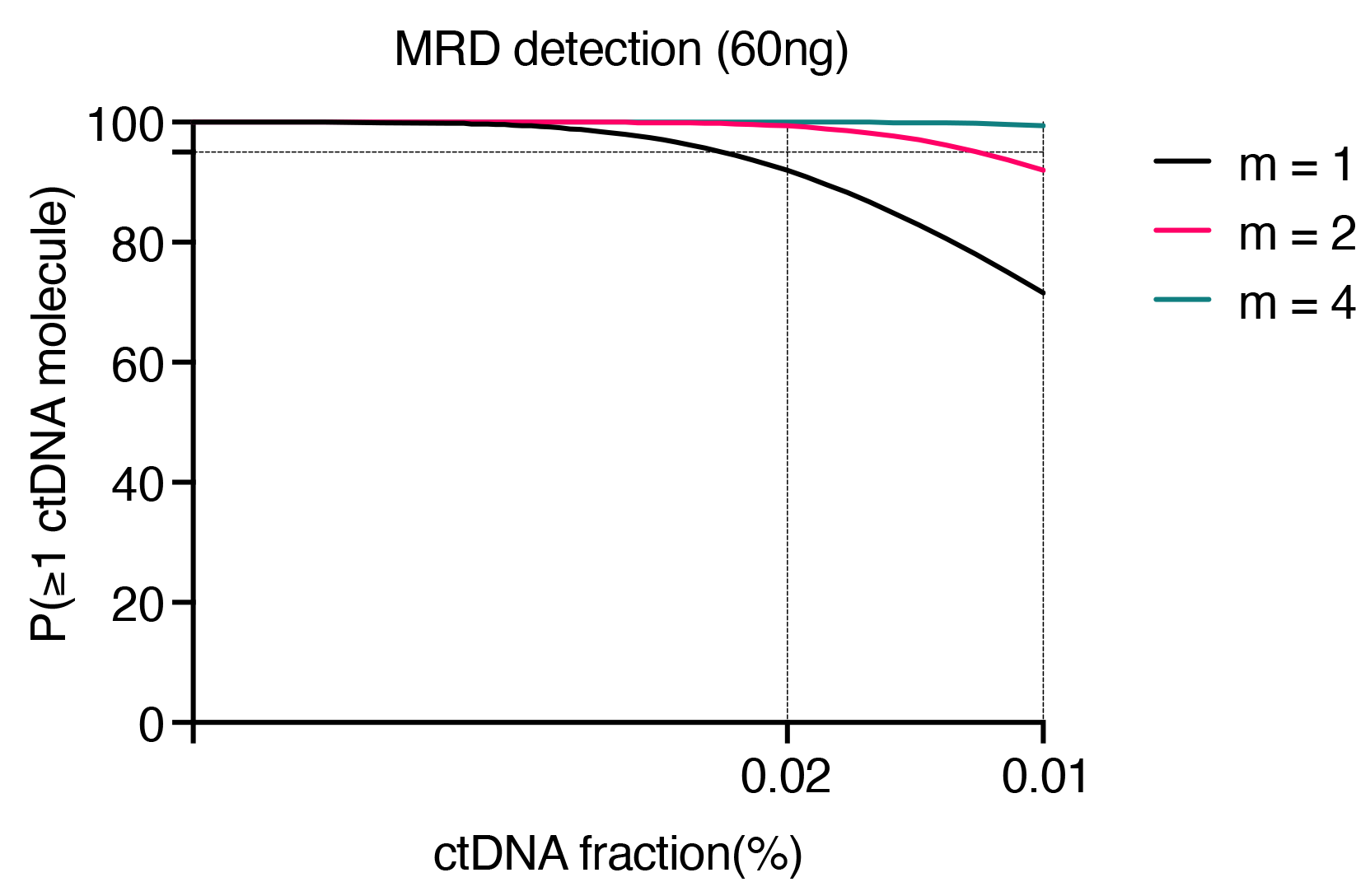

Supplement: S2 Fig — (TIF) [file pcbi.1013443.s003.tif]
